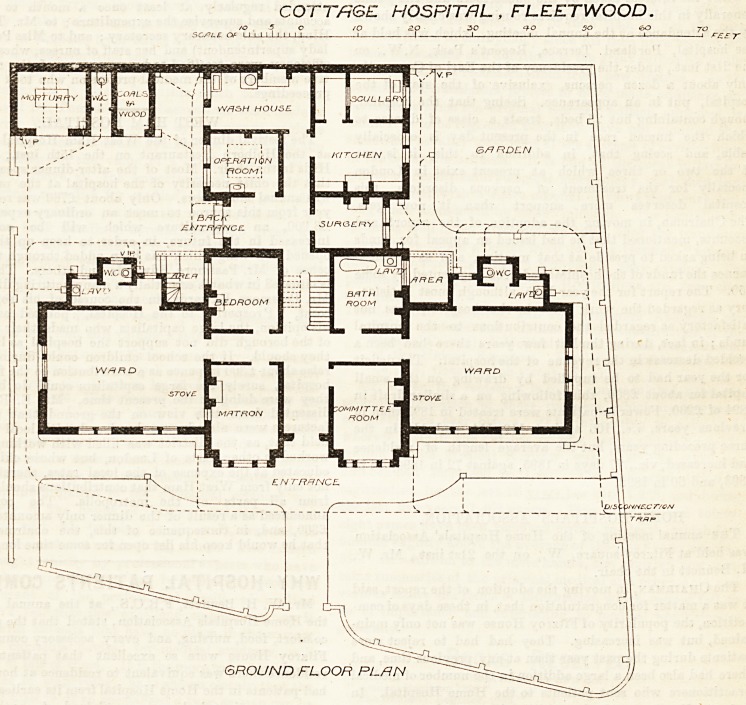# Hospital Construction

**Published:** 1896-04-25

**Authors:** 


### HOSPITAL CONSTRUCTION.

### FLEETWOOD COTTAGE HOSPITAL.

This hospital has been constructed with a view to relieving the pressure upon the resources of the infirmary at Preston, to which patients had previously to be conveyed. The scheme has been mooted for about three years, but has not taken definite shape until the present time. The plans are the work of Messrs. Drummond and Sons (who are also contractors for the building). The principal entrance, facing south, is placed between a matron's room and a committee room. Two large wards, measuring 28 feet by 22 feet, for four beds each, form wings to the central block. A bed-room and bath-room open off the corridor connecting the wards, and a staircase leads to the upper floor of the main block, where two small wards, three bed-rooms, a bath-room, and w.c. (both in one apartment) are provided. At right angles to the ward wings, and in rear of the central block, runs the kitchen wing. An operating-room and small surgery are provided in this portion of the building, but in proximity to the back entrance—an injudicious arrangement. A wash-house is placed at the end of a dark passage leading past the kitchen, and a mortuary and other offices in the yard, through which it would appear that tradesmen, and such patients as require treatment in the surgery or operating-room only, are intended to pass; this is undesirable, as interfering with the duties and privacy of the servants, and with a convenient connection between the kitchen and the hospital. The sanitary arrangements in connection with the wards consist of a w.c. and lavatory basin only, providing no sluice or sink, and are not cut off in the best manner, though this might easily have been effected by a cross ventilated lobby of the accepted kind. The proposed position of the beds in the wards is not indicated, nor does it appear to have been carefully studied with regard to windows, doors, and fireplaces. Portions of the passages would appear to be left in darkness, and the lighting and airing of a hospital staircase by a skylight only should have been avoided. Other defects in planning have been noted in the general description of the arrangements adopted, but no doubt the building will prove very useful in the locality.

COTTAGE HOSPITAL, FLEETWOOD.

GROUND FLOOR PLAN

## Figures and Tables

**Figure f1:**